# Complete characterization of RNA biomarker fingerprints using a multi-modal ATR-FTIR and SERS approach for label-free early breast cancer diagnosis†

**DOI:** 10.1039/d3ra05723b

**Published:** 2024-01-23

**Authors:** Shuyan Zhang, Steve Qing Yang Wu, Melissa Hum, Jayakumar Perumal, Ern Yu Tan, Ann Siew Gek Lee, Jinghua Teng, U. S. Dinish, Malini Olivo

**Affiliations:** a Institute of Materials Research and Engineering (IMRE), Agency for Science, Technology and Research (A*STAR) 2 Fusionopolis Way, Innovis #08-03 Singapore 138634 Republic of Singapore jh-teng@imre.a-star.edu.sg dinish@asrl.a-star.edu.sg malini_olivo@asrl.a-star.edu.sg; b Division of Cellular and Molecular Research, Humphrey Oei Institute of Cancer Research, National Cancer Centre Singapore (NCCS) 30 Hospital Boulevard Singapore 168583 Republic of Singapore dmslsg@nccs.com.sg; c Breast & Endocrine Surgery, Tan Tock Seng Hospital (TTSH) 11 Jln Tan Tock Seng Singapore 308433 Republic of Singapore; d Lee Kong Chian School of Medicine, Nanyang Technological University 50 Nanyang Avenue Singapore 639798 Republic of Singapore; e SingHealth Duke-NUS Oncology Academic Clinical Programme (ONCO ACP), Duke-NUS Medical School Singapore 169857 Republic of Singapore; f Department of Physiology, Yong Loo Lin School of Medicine, National University of Singapore Singapore 117593 Republic of Singapore

## Abstract

Breast cancer is a prevalent form of cancer worldwide, and the current standard screening method, mammography, often requires invasive biopsy procedures for further assessment. Recent research has explored microRNAs (miRNAs) in circulating blood as potential biomarkers for early breast cancer diagnosis. In this study, we employed a multi-modal spectroscopy approach, combining attenuated total reflection Fourier transform infrared (ATR-FTIR) and surface-enhanced Raman scattering (SERS) to comprehensively characterize the full-spectrum fingerprints of RNA biomarkers in the blood serum of breast cancer patients. The sensitivity of conventional FTIR and Raman spectroscopy was enhanced by ATR-FTIR and SERS through the utilization of a diamond ATR crystal and silver-coated silicon nanopillars, respectively. Moreover, a wider measurement wavelength range was achieved with the multi-modal approach than with a single spectroscopic method alone. We have shown the results on 91 clinical samples, which comprised 44 malignant and 47 benign cases. Principal component analysis (PCA) was performed on the ATR-FTIR, SERS, and multi-modal data. From the peak analysis, we gained insights into biomolecular absorption and scattering-related features, which aid in the differentiation of malignant and benign samples. Applying 32 machine learning algorithms to the PCA results, we identified key molecular fingerprints and demonstrated that the multi-modal approach outperforms individual techniques, achieving higher average validation accuracy (95.1%), blind test accuracy (91.6%), specificity (94.7%), sensitivity (95.5%), and *F*-score (94.8%). The support vector machine (SVM) model showed the best area under the curve (AUC) characterization value of 0.9979, indicating excellent performance. These findings highlight the potential of the multi-modal spectroscopy approach as an accurate, reliable, and rapid method for distinguishing between malignant and benign breast tumors in women. Such a label-free approach holds promise for improving early breast cancer diagnosis and patient outcomes.

## Abstract 2

With the multi-modal approach combining ATR-FTIR and SERS, we achieved an extended spectral range for molecular fingerprint detection of RNA biomarkers. Machine learning results shows 91.6% blind test accuracy for label-free breast cancer diagnosis.

## Graphical abstract

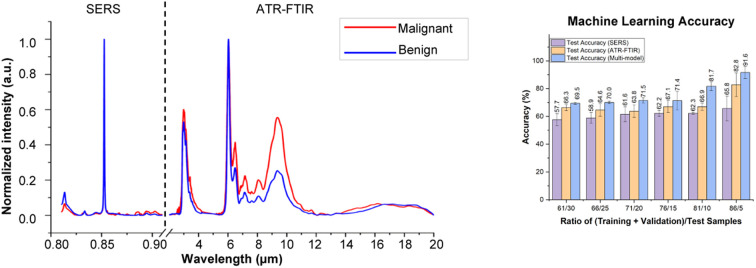

## 1. Introduction

Breast cancer is a significant global health concern and remains the most commonly diagnosed cancer in women worldwide. In 2020 alone, approximately 2.3 million new cases were reported, with a total of 7.8 million women living with breast cancer diagnosed over the past five years.^1^ Timely detection and treatment are crucial for improving survival rates. Although mammography, an X-ray imaging technique, serves as the current gold standard for breast cancer screening, it has limitations, with approximately 20% of breast cancer cases going undetected.^2,3^ Furthermore, the current mainstream diagnostic tools, including mammography, magnetic resonance imaging (MRI), and ultrasonography, often yield false positives, leading to undue stress for patients and additional diagnostic procedures.^3–6^ Therefore, there is an unmet clinical need for a rapid, accurate, and reliable test for breast cancer screening. One potential solution lies in the detection of biomarkers, specifically circulating microRNAs (miRNAs), which have shown promise in differentiating between individuals with and without cancer, particularly for those with abnormal mammograms.^7–9^

MiRNAs are small, non-coding RNA molecules, approximately 22 nucleotides in length. They have emerged as promising biomarkers for cancer detection due to their stability and abundance in body fluids such as serum and plasma.^10,11^ Unlike other RNA molecules, miRNAs possess specific structures that render them resistant to degradation by nucleases. This unique characteristic makes them attractive candidates for early cancer detection, as miRNA expression patterns have been found to be deregulated in cancer patients. Moreover, miRNAs exhibit wide distribution in various organs, indicating their potential utility in personalized medicine. Although existing detection techniques such as quantitative reverse transcriptase polymerase chain reaction (RT-qPCR) and next-generation sequencing (NGS) demonstrate high sensitivity and specificity,^12–14^ their utilization can be expensive and time-consuming due to the need for chemical labeling. Hence, there is a demand for faster and more affordable methods for miRNA detection.

Fourier transform infrared (FTIR) spectroscopy is a powerful tool for analyzing the chemical composition and molecular structure of biological samples. This technique measures the absorption of light by the sample, providing a molecular fingerprint that can detect changes associated with disease progression. The attenuated total reflection FTIR (ATR-FTIR) spectroscopy utilizes a high refractive index crystal. When infrared light is incident on the crystal, it creates an evanescent wave due to differences in refractive indices between the crystal and the sample. This means that only the molecules in close proximity to the crystal surface interact with the evanescent wave, leading to a stronger signal for a thin layer of the sample compared to traditional FTIR. ATR-FTIR spectroscopy has the potential for rapid and accurate detection of miRNAs for early cancer diagnosis and personalized medicine.^15–19^ Raman spectroscopy provides molecular information and can have sensitivity enhanced by surface-enhanced Raman scattering (SERS) to detect low-concentration samples.^20,21^ SERS utilizes nano-roughened surfaces coated with metal (like copper, silver, or gold), called planar SERS substrates, or metal colloidal nanoparticles to enhance the Raman signal, enabling the detection of miRNA fingerprints at very low concentrations. Both ATR-FTIR and SERS techniques are label-free techniques that have been used for the detection of biomolecules in various biological samples.^22–25^

Previous studies have demonstrated the potential of FTIR and SERS techniques for sensitive and accurate detection and analysis of nucleic acids.^26–28^ For instance, D. Li *et al.* and Y. Li *et al.* utilized SERS to detect miRNA and RNA bases, respectively, achieving improved sensitivity.^29,30^ Rios *et al.* employed FTIR spectroscopy to detect DNA polymorphisms with high accuracy using machine learning algorithms.^31^ Geinguenaud *et al.* utilized FTIR spectroscopy to study RNA structures, identifying key vibrational modes associated with RNA sugar puckering, backbone vibrations, phosphate stretching, and protein secondary structures.^32^ These studies underscore the potential of using spectroscopy techniques for sensitive and accurate detection and analysis of nucleic acids.

Concurrently, the integration of machine learning and chemometrics with spectroscopy has gained interest not just for medical diagnostics,^33–35^ but also for applications such as food quality control, detection of chloramphenicol in food products,^36^ and the comparative study of chemometric challenges in food analysis.^37^ The energy sector is similarly evolving with these methodologies. Progress in dye-sensitized solar cells is attributed to insights into interfacial effects in solid–liquid electrolytes,^38^ the effect of polymer electrolytes at the nanoscale,^39^ and the tuning of properties in carbazole photosensitizers.^40^ Supercapacitors, another essential energy storage technology, have also benefited from machine learning, as seen in the work on laser-induced graphene-based capacitors.^41^

In this paper, we present a novel multi-modal spectroscopy approach for early breast cancer diagnosis using combined ATR-FTIR and SERS data. Our study involved the measurement of 91 clinical samples with malignant and benign diagnoses previously confirmed through histopathology analysis. We explored a total of 32 machine learning models, each with varying training, validation, and blind test ratios, for the classification task using ATR-FTIR alone, SERS alone, and the combined multi-modal data. The results showed that the multi-modal approach achieved the best performance, with a validation accuracy of 95.1% and a test accuracy of 91.6%. Among the machine learning models, the support vector machine (SVM) outperformed others, demonstrating an impressive area under the curve (AUC) value of 0.9979. This outcome demonstrates that multi-modal spectroscopy provides complementary information and improves the accuracy of miRNA detection. Our label-free and rapid testing method, assisted by machine learning, offers a comprehensive characterization of the molecular fingerprints of biomarker molecules and high accuracy in early breast cancer diagnosis.

## 2. Materials and methodology

### 2.1. Samples

The sample collection and processing procedures are similar to our previous study.^19^ Serum samples for the analysis of microRNAs (miRNAs) were obtained from peripheral blood samples collected at the National Cancer Centre Singapore (Singapore) and Tan Tock Seng (Singapore) prior to biopsy and surgery. Additional serum samples were obtained from the SingHealth Tissue Repository (Singapore). These samples were not purchased or donated. The study followed the principles of the Declaration of Helsinki with approval from the Centralized Institutional Review Board of SingHealth (CIRB Ref: 2018/2874). Written informed consent was obtained from all participants.

A total of 91 samples were included in this study, with 44 diagnosed as malignant and 47 as benign based on histopathology analysis. To minimize the impact of confounding factors and technical biases in data analysis, pre-analytical factors, including sample collection, handling, processing, and storage, were standardized.^10^ Blood samples were collected and promptly processed within 50–60 minutes of venipuncture to separate serum from whole blood. The serum samples were aliquoted and stored at −80 °C to prevent freeze–thaw cycles, with only non-hemolyzed samples used in this study. Subsequently, total RNA was isolated from 200 μL of serum using the miRNeasy Serum/Plasma Advanced Kit (Qiagen, N.V.), following the manufacturer protocol. An additional step involving the addition of bacteriophage MS2 RNA to the sample lysis buffer (1 μg mL^−1^ of QIAzol) was included to enhance the RNA yield. Total RNA extraction was performed using the same reagents and procedures for all 91 samples.

### 2.2. Experimental setup

**Fig. 1:**
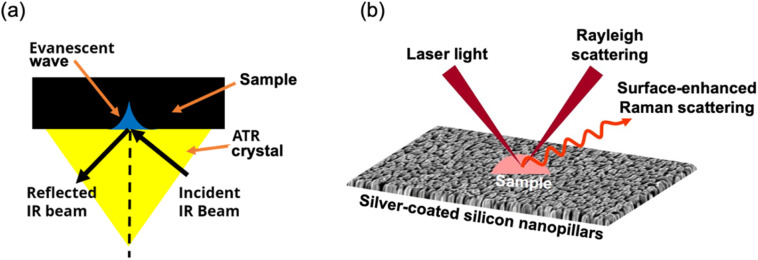
(a) ATR-FTIR spectroscopy uses a beam from a globar source entering an ATR crystal. Through internal reflection, an evanescent wave interacts with the sample, absorbing specific infrared frequencies. The reflected beam, carrying this information, is then directed to the FTIR detector. (b) SERS uses laser light to detect enhanced scattered photons *via* plasmonic effects. The sample is deposited on nano-roughened, metal-coated surfaces (SERS substrates). Interaction with laser light amplifies the Raman signal due to the electric field from silver-coated nanopillars, offering detailed molecular insights for precise sample detection.

The study employed ATR-FTIR and SERS techniques to analyze miRNA samples for early breast cancer diagnosis. ATR-FTIR spectroscopy, as illustrated in Fig. 1(a), utilizes an incident beam from a globar source that enters an ATR crystal with a high refractive index. Through total internal reflection, the beam is reflected at the crystal–sample interface, creating an evanescent wave that penetrates the sample. During this interaction, specific frequencies of light in the infrared range are absorbed by the sample, resulting in characteristic absorption bands. The reflected beam carries the spectral information of the absorbed frequencies and is directed toward the FTIR detector. ATR-FTIR spectroscopy provides valuable insights into the molecular composition and interactions within the sample, making it a powerful analytical technique for various applications. In this study, an ATR-FTIR system (Vertex 80v with ATR diamond crystal accessory, Bruker) was used to obtain spectra from 10 μL of miRNA samples under a vacuum condition. Each clinical sample was subjected to 20 measurements sequentially without changing the sample. For each measurement, an average was taken based on 64 scans at a resolution of 4 cm^−1^. All these measurement results were then used for subsequent analysis. The vacuum condition ensured that the collected data was free from interference by water vapour,^19^ as shown in the ESI, Fig. S1.†

As depicted in Fig. 1(b), SERS involves the illumination of the sample with laser light and the detection of the enhanced inelastically scattered photons through the plasmonic effect. Enhancement of Raman signal is achieved by depositing the sample on nano-roughened metal-coated surfaces called SERS substrates. Here, SERS substrates were fabricated on silicon wafers, and nanostructures were in the form of nanopillars, which were formed using the inductively coupled plasma-based blanket etching method. The size of nanopillars was typically ∼200 nm in height, and it was coated with a 150 nm layer of silver.^42^ When the laser light interacts with the sample, due to the localized electric field enhancement generated by the silver-coated nanopillars, resulting in amplifying the Raman signal of the molecules in the proximity. This enhanced Raman scattering provides detailed molecular information, enabling sensitive and selective detection of the sample. SERS offers immense potential for various applications, including chemical analysis and biosensing.^43^ SERS measurements were conducted using a Raman microscope system (Invia, Renishaw) integrated with a Leica microscope. The laser light (785 nm) was coupled through a long working distance objective lens (50×, 0.5 NA) to excite the sample and collect the scattered Raman signal. The clinical miRNA samples (10 μL) were pipetted onto the bare SERS substrates, and enhanced Raman signals were collected in backscattering geometry. Multiple measurements were taken at 20 different locations (∼20 μm apart) on the substrate, and averaged spectra were used for analysis. The spectral measurements were performed with a laser power of ∼450 μW.

### 2.3. Data processing workflow

**Fig. 2:**
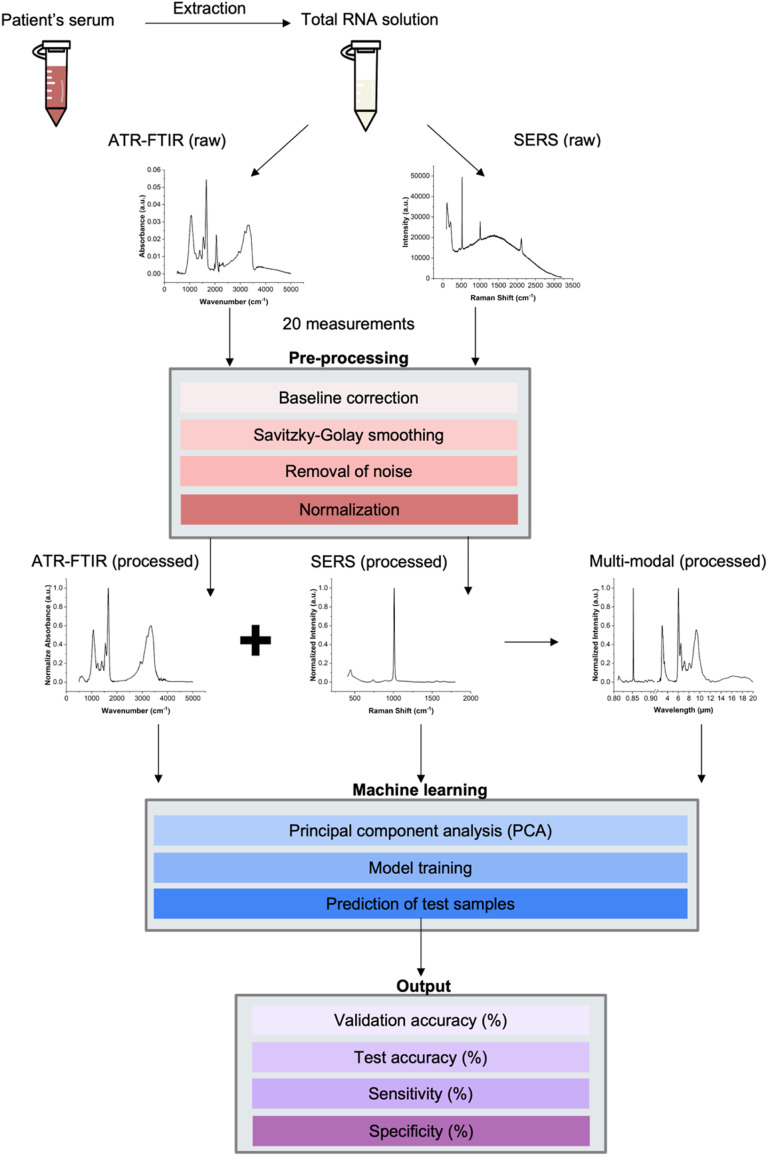
Workflow illustrating the process of sample preparation; data collection using ATR-FTIR and SERS techniques; data processing for ATR-FTIR alone, SERS alone, and multi-modal; machine learning, and final output including validation accuracy, test accuracy, sensitivity, and specificity.

The workflow of the sample preparation, data collection, and data analysis is illustrated in Fig. 2. The raw data underwent pre-processing steps before machine learning analysis, which included baseline correction, Savitzky–Golay smoothing, removal of noisy and atmospheric peaks, and normalization. The processed ATR-FTIR and SERS data were combined based on the wavelength. ATR-FTIR wavenumber was converted to the wavelength using eqn (1), and the SERS Raman shift was converted to the wavelength using eqn (2), where *λ*_ex_ = 785 nm. After the conversion, the ATR-FTIR data ranged from 2 to 20 μm, and SERS data ranged from 0.8 to 0.9 μm. Consequently, the combined multi-modal data spanned from 0.8 to 20 μm with a gap of 1.1 μm from 0.9 to 2 μm.1
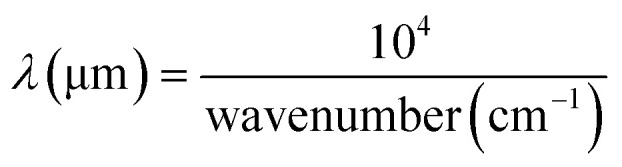
2
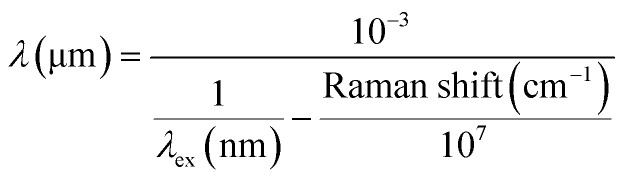


Machine learning algorithms were applied to the processed ATR-FTIR data, SERS data, and multi-modal data separately. The steps included principal component analysis (PCA), model training, and prediction of test results. The outcomes were evaluated using five parameters: validation accuracy, test accuracy, sensitivity, specificity, and *F*-score.

### 2.4. Machine learning methods

In this study, a total of 32 different machine learning models were developed and trained using MATLAB (R2022a, MathWorks). During the model training process, PCA and cross-validation methods were implemented to enhance the accuracy and robustness of the models.

#### 2.4.1. Data preparation

The dataset consisting of spectroscopic measurements of 91 samples was divided into training and test datasets. To assess the model performance, 6 different sets of blind test samples (*i.e.*, not overlapping with the training and validation datasets) were selected, including 5, 10, 15, 20, 25, and 30 test samples. The remaining samples were utilized for training and validation purposes to construct the machine learning models. Table 1 provides a breakdown of the sample splitting, indicating the ratio of test samples to training + validation samples. To eliminate potential biases in the test dataset, each ratio was run three times, with each run employing a randomly selected test sample set.

**Table 1:** Breakdown of sample splitting for machine learning datasets with different test *vs.* training + validation ratios. The total number of samples is 91, with 44 being malignant and 47 being benign samples

Ratio	Run	Training + validation samples			Test samples			Total samples		
		Malignant	Benign	Total	Malignant	Benign	Total	Malignant	Benign	Total
0.058	1	41	45	86	3	2	5	44	47	91
	2	43	43		1	4				
	3	42	44		2	3				
0.123	1	39	42	81	5	5	10			
	2	41	40		3	7				
	3	40	41		4	6				
0.197	1	36	40	76	8	7	15			
	2	37	39		7	8				
	3	38	38		6	9				
0.282	1	33	38	71	11	9	20			
	2	34	37		10	10				
	3	34	37		10	10				
0.379	1	31	35	66	13	12	25			
	2	31	35		13	12				
	3	32	34		12	13				
0.492	1	28	33	61	16	14	30			
	2	29	32		15	15				
	3	29	32		15	15				

Ten-fold cross-validation and PCA were employed for training the models. Ten-fold cross-validation involved partitioning the dataset into ten sets of data, with one set used for validation and the other sets utilized for training. This methodology ensured that the models were trained on different datasets, promoting greater generalization and robustness. As the spectral data used in this study had high dimensionality, PCA was employed to reduce the computational requirements. The Origin software (2022a, OriginLab) was utilized to perform PCA by generating a scree plot and identifying the elbow point to determine the optimal number of principal components (PCs). PCA was conducted for each data method (ATR-FTIR, SERS, and Multi-modal).

#### 2.4.2. Types of models

The machine learning algorithms used in this study encompassed decision trees, discriminant analysis, logistic regression, naïve Bayes, SVM, *k*-nearest neighbors (KNN), ensemble models, neural networks, and kernel approximations. A list of the models is provided in the ESI, Table S1.† The selection of these models allowed for a comparison of their performance on different datasets. Decision trees utilize conditions to make decisions and branch into different branches based on predictor values and trained weights. Discriminant analysis classifies data based on Gaussian distributions, while logistic regression employs a sigmoid curve as a decision boundary. Naïve Bayes classifiers utilize the Bayes theorem to calculate the probability of a sample belonging to a particular class. SVMs utilize separating hyperplanes to distinguish data points, and KNN models classify samples based on the classes of their nearest neighbors. Ensemble models combine weaker techniques such as bagging and boosting to create a more robust ensemble model. Neural networks consist of layers of neurons with weights that are trained during model training, while kernel approximations transform lower-dimensional data into higher-dimensional data using kernel functions, enabling linear classifiers such as planes to separate data points belonging to different classes.

#### 2.4.3. Model selection

The performance of the models was evaluated using various metrics, including validation and test accuracy, the discrepancy between the validation and test accuracy, specificity, sensitivity, and *F*-score. Models with a discrepancy exceeding 15% were excluded to prevent overfitting. These metrics were calculated using the true positive (TP), false positive (FP), true negative (TN), and false negative (FN) values obtained from the confusion matrix, as shown in eqn (3)–(5).3
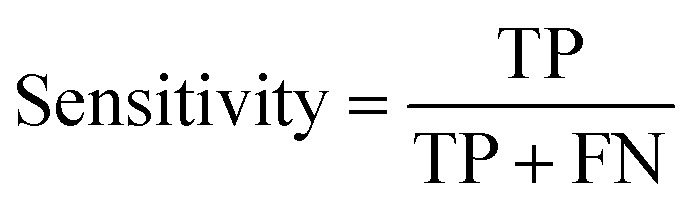
4
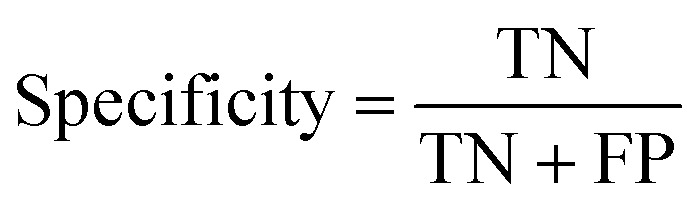
5
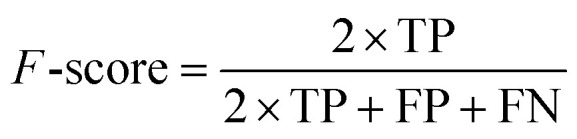


## 3. Results and discussion

### 3.1. Molecular fingerprints

**Fig. 3:**
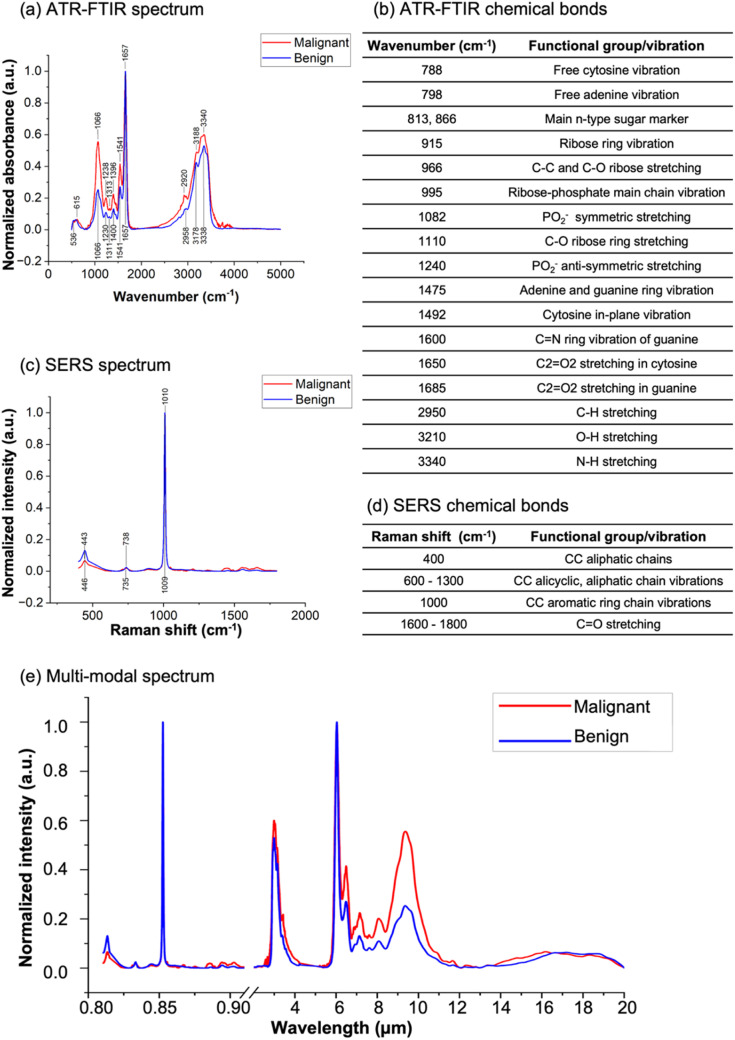
Molecular fingerprint measurements of malignant (red curves) and benign (blue curves) samples. ATR-FTIR: (a) normalized average spectrum with labeled peak wavenumbers and (b) corresponding chemical bonds. Two distinct fingerprint regions are observed: one ranging from 500 to 2000 cm^−1^ and the other from 2500 to 3500 cm^−1^. SERS: (c) normalized average spectrum with labeled peak wavenumbers and (d) corresponding chemical bonds. Multi-modal: (e) average spectrum of ATR-FTIR and SERS where the ATR-FTIR wavenumber units and SERS Raman shift units were converted to wavelength units based on eqn (1) and (2).

Fig. 3 presents the average of the measurement results obtained using both ATR-FTIR and SERS techniques. Fig. 3(a) displays the normalized absorption spectra of a malignant sample (red) and a benign sample (blue) as measured by ATR-FTIR spectroscopy. Two distinct fingerprint regions are observed: one ranging from 500 to 2000 cm^−1^ and the other from 2500 to 3500 cm^−1^. This characteristic is consistent across all samples. To validate our measurement accuracy and reproducibility, we also measured synthetic miRNA samples, observing similar features as shown in the ESI, Fig. S2.† Additionally, it can be noted that the peak wavenumbers are nearly identical for both sample types, but the relative peak intensities differ. For instance, the differences in peak intensities at 1066 cm^−1^, 1541 cm^−1^, and 3340 cm^−1^ are smaller for the malignant samples compared to the benign samples. Moreover, the width of the broad peak from 2500 to 3500 cm^−1^ is larger for the malignant samples than for the benign samples. Fig. 3(b) illustrates the peak wavenumbers and their corresponding chemical bonds and vibrational groups for DNA and RNA molecules, as documented in the literature.^32,44,45^ The most prominent peak wavenumber in both malignant and benign spectra is observed at 1657 cm^−1^, corresponding to C2=O2 stretching in cytosine or guanine. The second notable peaks are located at 3188 cm^−1^ and 3340 cm^−1^, corresponding to O–H stretching and N–H stretching, respectively. It is worth mentioning that the peak intensity at 1066 cm^−1^ is more pronounced in malignant samples than in benign samples, corresponding to PO_2_^−^ symmetric stretching.

On the other hand, the SERS spectra in Fig. 3(c) reveal limited molecular fingerprints. The most prominent peak is observed at 1010 cm^−1^, accompanied by a small peak at 446 cm^−1^. The functional groups associated with these peaks are depicted in Fig. 3(d), with 1010 cm^−1^ representing CC aromatic ring chain vibrations and 446 cm^−1^ indicating CC aliphatic chains.

Fig. 3(e) showcases the multi-modal spectra. The smaller wavelength region represents the SERS spectra, while the larger wavelength region represents the ATR-FTIR spectra. Notably, after the wavelength conversion, the ATR-FTIR spectra were horizontally flipped. It is evident that the number of SERS peaks is considerably lower than that of the ATR-FTIR peaks.

### 3.2. Visual peak analysis

**Fig. 4:**
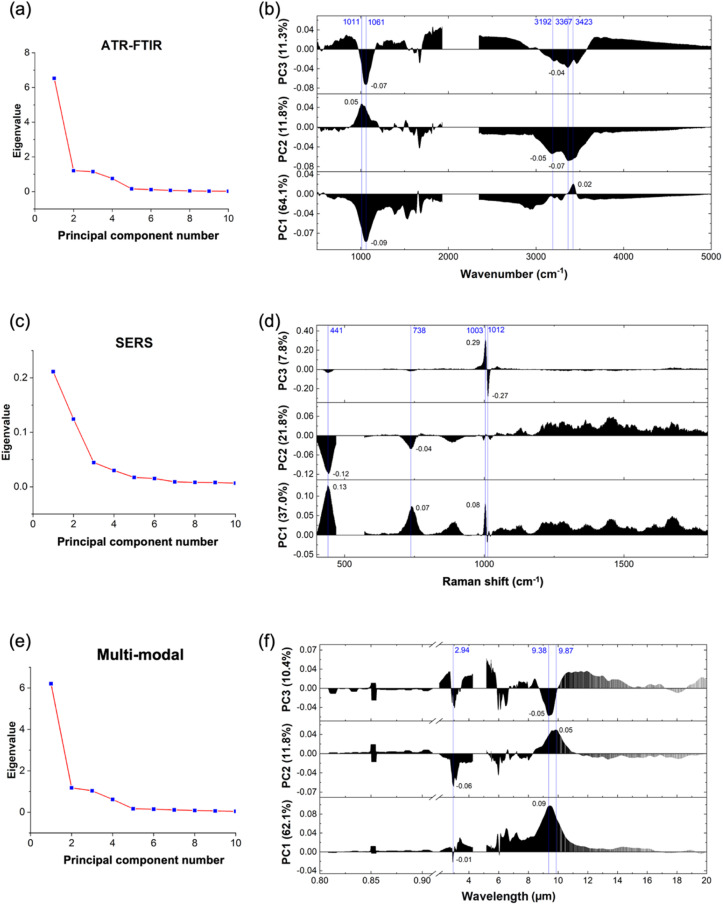
Results of principal component analysis. ATR-FTIR data: (a) scree plot indicating the third point is the elbow point and (b) loading with reference wavenumber plot showing PC1, PC2, and PC3 characteristic wavenumbers (marked in blue) and their corresponding loading values (marked in black). SERS data: (c) scree plot indicating the sixth point is the elbow point and (d) loading with reference wavenumber plot showing PC1, PC2, and PC3 characteristic wavenumbers (marked in blue) and their corresponding loading values (marked in black). Multi-modal data: (e) scree plot indicating the third point is the elbow point and (f) loading with reference wavenumber plot showing PC1, PC2, and PC3 characteristic wavenumbers (marked in blue) and their corresponding loading values (marked in black). It is shown that the ATR-FTIR data dominate in the characteristics than the SERS data in the multi-modal approach.

PCA is a powerful approach for reducing and interpreting large multivariate datasets with linear structures, enabling the discovery of previously unsuspected relationships. In this study, PCA was applied to the ATR-FTIR, SERS, and multi-modal data, as depicted in Fig. 4. By utilizing PCA, we were able to investigate the relationship between the light absorption and scattering intensities of biomolecules and their respective wavelengths, while also determining the optimal number of PCs to retain. A scree plot, serving as a visual aid, was employed to identify the appropriate number of PCs. The number is determined by locating the “elbow” point where the remaining eigenvalues become relatively small and of comparable size.

In Fig. 4(a), the scree plot for the ATR-FTIR data is presented. Although the elbow point is not distinctly apparent, we consider the third point as the elbow point. Fig. 4(b) illustrates the loading with reference wavenumber plot for the ATR-FTIR data, showcasing the loading patterns of PC1, PC2, and PC3. These PCs collectively account for 87.2% of the total variance, with PC1 contributing 64.1%, PC2 contributing 11.8%, and PC3 contributing 11.3%. The vertical lines on the plot indicate the important wavenumbers for each PC. Notably, PC1 is associated with significant wavenumbers at 1061 cm^−1^ and 3423 cm^−1^, with respective loading values of −0.09 and 0.02. For PC2, the influential wavenumbers include 1011 cm^−1^, 3192 cm^−1^, and 3367 cm^−1^, with corresponding loading values of 0.05, −0.05, and −0.07. In PC3, the crucial wavenumbers are 1061 cm^−1^ and 3367 cm^−1^, with respective loading values of −0.07 and −0.04. These findings align with the spectra presented in Fig. 3(a), where 1061 cm^−1^ corresponds to PO_2_^−^ symmetric stretching and 3367 cm^−1^ corresponds to N–H stretching.^46,47^ Notably, the important wavenumbers for each PC correspond to specific chemical bonds or functional groups that are significant in differentiating between malignant and benign samples. These chemical bonds or functional groups play a vital role in DNA and RNA structures, and their variation can provide insights into the differences between malignant and benign DNA/RNA solutions.^48^

For the SERS data, Fig. 4(c) showcases the scree plot, indicating the sixth point as the elbow point. Fig. 4(d) presents the loading with reference Raman shift plot for the SERS data, illustrating the loadings of the first three PCs. PC1 accounts for 37.0% of the total variance, PC2 for 21.8%, and PC3 for 7.8%. The vertical lines on the plot correspond to important Raman shifts for each PC. PC1 is characterized by significant Raman shifts at 441 cm^−1^, 738 cm^−1^, and 1003 cm^−1^, with respective loading values of 0.13, 0.07, and 0.08. In PC2, the influential features include 441 cm^−1^ and 738 cm^−1^, with loading values of −0.12 and −0.04, respectively. PC3 is characterized by the prominent Raman shifts at 1003 cm^−1^ and 1012 cm^−1^, with respective loading values of 0.29 and −0.27. Notably, the Raman shifts at 441 cm^−1^ and 738 cm^−1^ are important in both PC1 and PC2, while 1003 cm^−1^ exhibits more influence in PC1 and PC3. These findings are consistent with the spectra depicted in Fig. 3(c), where 441 cm^−1^ corresponds to CC aliphatic chains, 738 cm^−1^ is likely due to CC alicyclic and aliphatic chain vibrations, and 1003 cm^−1^ may be associated with aromatic ring chain vibrations. These molecular features are relevant to DNA and RNA structures and exhibit variations that contribute to the distinction between malignant and benign samples.^49^

Fig. 4(e) displays the scree plot for the multi-modal data, with the third point identified as the elbow point. Fig. 4(f) illustrates the loading with reference wavelength for the multi-modal data, highlighting the contributions of the first three PCs. PC1 accounts for 62.1% of the total variance, PC2 for 11.8%, and PC3 for 10.4%. The vertical lines on the plot denote the important wavelengths for each PC. Notably, 2.94 μm is a significant wavelength in both PC1 and PC2, while 9.38 μm exhibits more influence in PC1 and PC3. These findings align with the spectra depicted in Fig. 3(e). Importantly, it is worth noting that all the significant features originate from the ATR-FTIR data region. This observation suggests that the ATR-FTIR technique is notably more efficient than the SERS technique in classifying malignant and benign breast cancer miRNA biomarkers. More advanced SERS techniques may be explored to improve its detection efficiency, such as introducing an interfacial agent or aggregating agent.^29,30^

In summary, the application of PCA to the ATR-FTIR, SERS, and multi-modal data provides valuable insights into the relationships between biomolecular absorption or scattering intensities and their corresponding wavenumbers or wavelengths. By identifying important wavenumbers and wavelengths associated with specific chemical bonds or functional groups, PCA enables the differentiation between malignant and benign miRNA solutions, contributing to the classification of breast cancer biomarkers.

### 3.3. Machine learning results

**Fig. 5:**
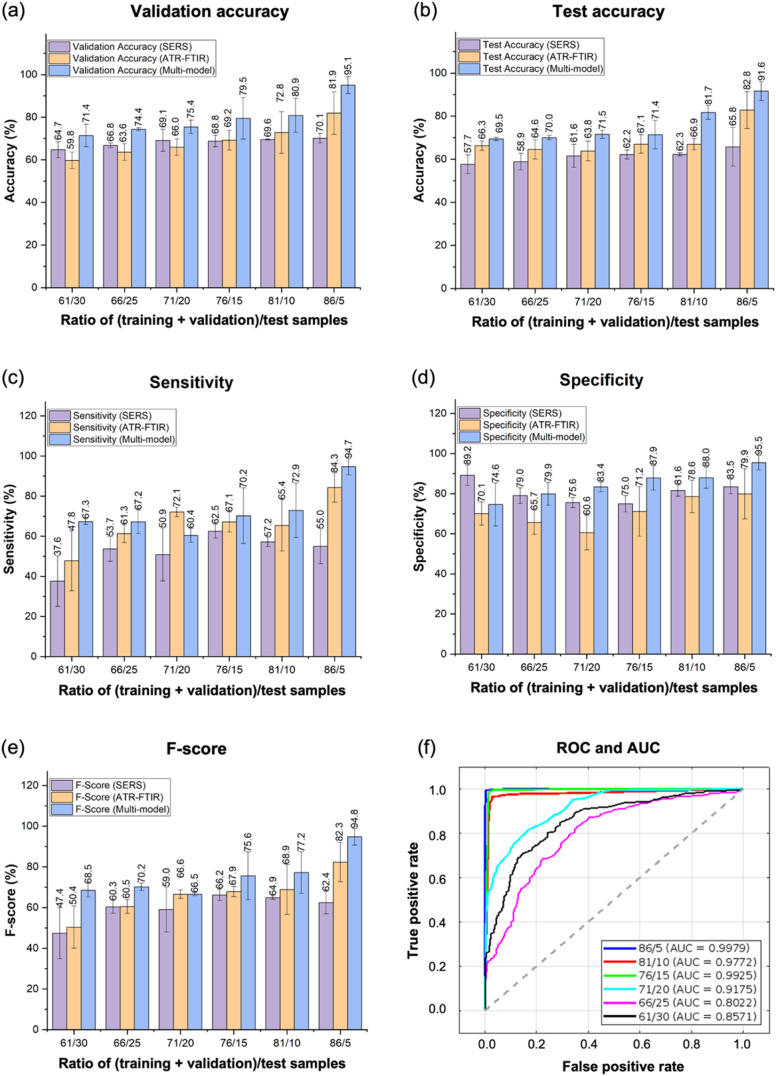
Machine learning results for various ratios of (training + validation)/test samples. Plots of (a) validation accuracy, (b) test accuracy, (c) sensitivity, (d) specificity and (e) *F*-score where SERS data is shown in purple, ATR-FTIR data is shown in orange, and multi-modal data is shown in blue. The numbers on the bar plots indicate the average values of the three runs and the error bars indicate the standard deviation. (f) Plots of the ROC curves (solid lines) and AUC values (legend values) for the multi-modal data for each ratio.

In this section, we will discuss the results and analysis of the machine learning models developed for breast cancer diagnosis using spectral data from three different methods – ATR-FTIR, SERS, and multi-modal spectroscopy. The selection criterion for choosing the best model for each dataset was based on high validation and test accuracies, small validation-test accuracy discrepancy, high sensitivity, specificity, and *F*-score. The average value and standard error were calculated across different runs for each ratio of (training + validation)/test samples of each measurement method, and these values were used for plotting, as shown in Fig. 5.

Fig. 5(a) and (b) depict the validation and test accuracy results, respectively. The average values of the three runs for each split ratio are represented on the bar plots, with standard deviations shown as error bars. The multi-modal data approach exhibits the highest validation accuracy, reaching an impressive 95.1%. With a validation accuracy of 95.1%, we can anticipate approximately 95 correct predictions out of every 100 samples tested. This accuracy level is comparable to the histopathology diagnosis with artificial intelligence assistance and surpasses the histopathology diagnosis alone, indicating its potential as a superior diagnostic tool.^50–52^ Notably, as the ratio increases, the accuracy also demonstrates improvement. However, even with a low ratio, a consistently high test accuracy of 69.5% is maintained. It is important to highlight that the SERS accuracy exhibits greater variations from the expected increasing trend, which can be attributed to the relatively fewer features present in the SERS data compared to ATR-FTIR and the multi-modal data. Moreover, the SERS accuracy generally tends to be lower than the ATR-FTIR accuracy, while the multi-modal accuracy surpasses both individual accuracies. This disparity can be explained by the additional information provided by the multi-modal spectroscopy data, which enhances the accuracy of the diagnostic predictions.

Fig. 5(c)–(e) present the results of the sensitivity, specificity, and *F*-score analyses. The multi-modal approach outperforms the ATR-FTIR and SERS data methods individually, achieving the highest sensitivity, specificity, and *F*-score, all at an impressive value of around 95%. This signifies the model ability to accurately classify 95 out of 100 true positive and true negative samples. Moreover, an increase in the ratio leads to improved sensitivity, specificity, and *F*-score. Notably, even at a low ratio, a consistently high sensitivity, specificity, and *F*-score of approximately 70% are maintained. It is important to note that the SERS data exhibits a less discernible trend in sensitivity, specificity, and *F*-score values. This behavior can be attributed to the relatively fewer features available in the SERS spectra, potentially limiting the model ability to capture the differential features required for distinguishing between malignant and benign classes. In addition, we have identified that the best models are the SVM, KNN, and SVM for ATR-FTIR, SERS, and multi-modal data methods, respectively.

Fig. 5(f) displays the receiver operating characteristic (ROC) curves and corresponding AUC values for the multi-modal data at each ratio. The color code is indicated in the legend. A perfect classifier would exhibit a true positive rate (sensitivity) of 1.0 and a false positive rate (1-specificity) of 0.0, while a random classifier is represented by the dashed line. The AUC value ranges from 0.0 to 1.0, with 1.0 indicating a perfect model. Our best AUC value of 0.9979 is achieved at the (training + validation)/test ratio of 86/5 using SVM, and the value generally decreases as the number of test samples increases, with the exception of the 76/15 ratio. Notably, even at the 61/30 ratio, our results demonstrate a relatively high AUC of 0.8571. These findings suggest promising discrimination capabilities in distinguishing between malignant and benign samples.

## 4. Conclusions

In conclusion, this study highlights the potential of utilizing the multi-modal spectroscopy approach for the detection of miRNA biomarkers in early breast cancer diagnosis. By combining the highly sensitive ATR-FTIR and SERS techniques, complete fingerprint profiles of the biomarkers were obtained. Notably, the ATR-FTIR technique provided a broader range of fingerprint profiles across a wider wavelength range compared to SERS. Machine learning analysis demonstrated the highest accuracy (95.1%) in classifying malignant and benign cases when utilizing the multi-modal approach. These findings indicate the effectiveness of the proposed approach for accurate and reliable label-free breast cancer diagnosis. Furthermore, the approach can be generalized to other biomarker types, including proteins and lipids, thereby expanding its potential applications in various areas of biomedical research. Overall, this study contributes to the development of a robust and versatile spectroscopy-based approach for early cancer detection and holds promise for future advancements in the field.

## Author contributions

Conception: S. Zhang, A. S. G. Lee, J. Teng, D. U. S., and M. Olivo. Clinical samples: M. Hum, E. Y. Tan, and A. S. G. Lee. Experiment: S. Zhang, Q. Y. S. Wu, and J. Perumal. Data analysis: S. Zhang. Manuscript preparation: all authors. Supervision: A. S. G. Lee, J. Teng, D. U. S., and M. Olivo.

## Conflicts of interest

There are no conflicts to declare.

## Supplementary Material

RA-014-D3RA05723B-s001
