# Supplementary material for: Complete characterization of RNA biomarker fingerprints using a multi-modal ATR-FTIR and SERS approach for label-free early breast cancer diagnosis
Source: RSC Adv. 2024 Jan 23;14(5):3599–610. doi: 10.1039/d3ra05723b (PMC10804230; doi:10.1039/d3ra05723b)
Supplement: RA-014-D3RA05723B-s001 [file RA-014-D3RA05723B-s001.pdf]

Electronic Supplementary Material (ESI) for RSC Advances.
This journal is © The Royal Society of Chemistry 2024

Supplementary Information
for
Complete Characterization of RNA Biomarker Fingerprints Using a Multi-Modal ATR-FTIR and SERS Approach for Label-Free Early Breast Cancer Diagnosis

1. ATR-FTIR measurements under vacuum and atmospheric conditions

Fig. S1(a) and (b) show the measurement results of DNA, miRNA, and water samples in the liquid form under atmospheric and vacuum conditions, respectively. Water has strong absorption in the infrared region; hence, the absorption peaks of DNA and miRNA molecules were entirely covered by water. With the vacuum process removing the water from the DNA and miRNA solution samples, the absorption peaks of DNA and miRNA molecules could then be visualized.

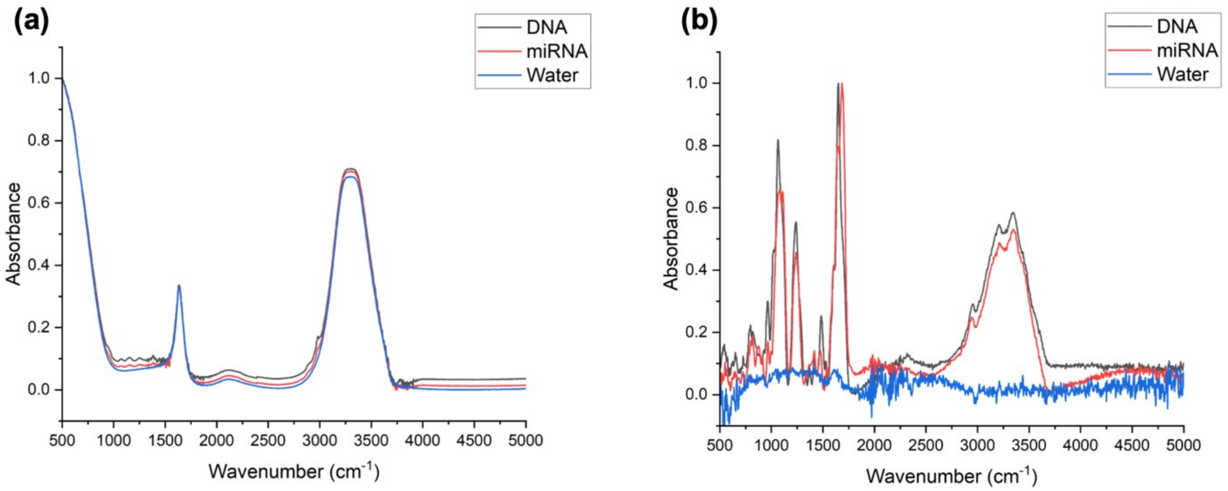

Figure S1. ATR-FTIR measurements of DNA (black curves), miRNA (red curves), and water (blue curves) solution samples under (a) atmospheric conditions and (b) under vacuum conditions.

2. Additional ATR-FTIR measurements on synthetic miRNA samples

Fig. S2 shows the measured spectra of synthetic miRNA samples under the vacuum condition. The results match with the clinical extracted samples with the prominent fingerprint regions broadly located in two regions, 800 – 2000 cm^{-1} and 2800 – 3500 cm^{-1}.

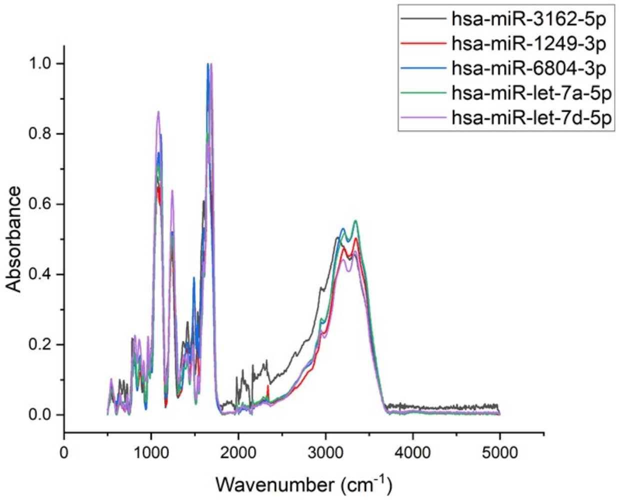

Figure S2. Measurement results of synthetic miRNA samples, hsa-miR-3162-5p (black curve), hsa-miR-1249-3p (red curve), hsa- miR-6804-3p (blue curve), hsa-miR-let-7a-5p (purple curve), and hsa-miR-let-7d-5p (yellow curve).

3. Machine learning models

Table S1 gives a list of the machine learning models that were studied in this manuscript.

Table S1. List of machine learning models

No.	Group	Model
1	Decision trees	Fine tree
2		Medium tree
3		Coarse tree
4		All trees
5		Optimizable tree
6	Discriminant analysis	Linear discriminant analysis
7		Quadratic discriminant analysis
8		Optimizable discriminant analysis
9	Logistic regression classifiers	Logistic regression
10	Naïve Bayes classifiers	Gaussian naive Bayes
11		Kernel naive Bayes
12		Optimizable naive Bayes
13	Support vector machines	Linear SVM
14		Quadratic SVM
15		Cubic SVM
16		Fine Gaussian SVM
17		Medium Gaussian SVM
18		Coarse Gaussian
19		Optimizable SVM
20	Nearest neighbor classifiers	Fine KNN
21		Medium KNN
22		Coarse KNN
23		Cosine KNN
24		Cubic KNN
25		Weighted KNN
26		Optimizable KNN
27	Ensemble classifiers	Boosted trees
28		Bagged trees
29		Subspace discriminant
30		Subspace KNN
31		Random undersampling boosted trees
32		Optimizable ensemble
